# Farnesal-loaded pH-sensitive polymeric micelles provided effective prevention and treatment on dental caries

**DOI:** 10.1186/s12951-020-00633-2

**Published:** 2020-06-11

**Authors:** Youping Yi, Lujun Wang, Lin Chen, Yan Lin, Zhongling Luo, Zhenyu Chen, Ting Li, Jianming Wu, Zhirong Zhong

**Affiliations:** Department of Pharmaceutical Sciences, School of Pharmacy, Southwest Medical University, Luzhou, Sichuan 646000 China

**Keywords:** pH-sensitive, Polymeric micelles, Hydroxyapatite, Farnesal, Dental caries

## Abstract

**Background:**

Farnesol is a sesquiterpene from propolis and citrus fruit that shows promising anti-bacterial activity for caries treatment and prevention, but its hydrophobicity limits the clinical application. We aimed to develop the novel polymeric micelles (PMs) containing a kind of derivative of farnesol and a ligand of pyrophosphate (PPi) that mediated PMs to adhere tightly with the tooth enamel.

**Results:**

Farnesal (Far) was derived from farnesol and successfully linked to PEG via an acid-labile hydrazone bond to form PEG-hyd-Far, which was then conjugated to PPi and loaded into PMs to form the aimed novel drug delivery system, PPi-Far-PMs. The in vitro test about the binding of PPi-Far-PMs to hydroxyapatite showed that PPi-Far-PMs could bind rapidly to hydroxyapatite and quickly release Far under the acidic conditions. Results from the mechanical testing and the micro-computed tomography indicated that PPi-Far-PMs could restore the microarchitecture of teeth with caries. Moreover, PPi-Far-PMs diminished the incidence and severity of smooth and sulcal surface caries in rats that were infected with *Streptococcus mutans* while being fed with a high-sucrose diet. The anti-caries efficacy of free Far can be improved significantly by PPi-Far-PMs through the effective binding of it with tooth enamel via PPi.

**Conclusions:**

This novel drug-delivery system may be useful for the treatment and prevention of dental caries as well as the targeting therapy of anti-bacterial drugs in the oral disease.
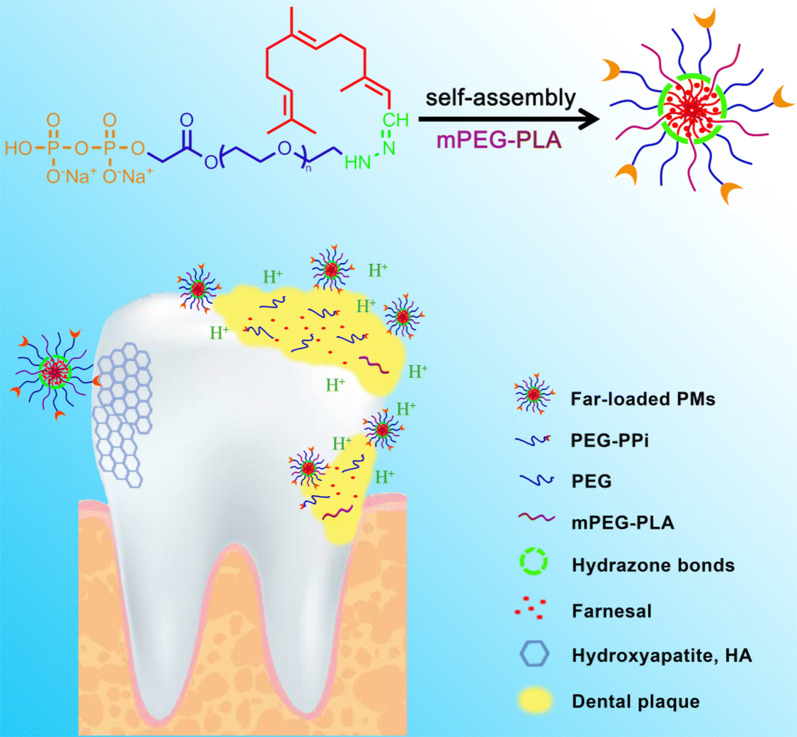

## Introduction

Dental caries is one of the most prevalent preventable diseases, and its incidence is especially high among young people. Caries progress when bacteria metabolize fermentable carbohydrates to produce acid, which dissolves the hydroxyapatite in teeth [1]. One promoter of caries progression is dental plaque, the complex multispecies biofilm formed by *Streptococcus mutans* and other organisms that colonize the surface of teeth [2, 3]. *S. mutans* can efficiently convert dietary sucrose into extracellular polysaccharides that promote *S. mutans* accumulation and form a dense matrix that protects the embedded bacteria [4, 5]. *S. mutans* ferments sucrose within the polysaccharide matrix, and creates a highly acidic microenvironment [6–8], with pH falling as low as pH 4.5 [9]. This acidity dissolves the enamel, initiating caries, which can lead to tooth loss, pain and infections [10–12].

Broad-spectrum antimicrobials such as chlorhexidine and triclosan can kill or inhibit the growth of cariogenic bacteria, but their poor selectivity means that they may disturb the normal balance of microflora in the oral cavity. Chlorhexidine may also stain teeth and cause calculus formation [10]. Fluoride products can prevent dental caries through promoting the remineralization process, but they carry the risk of dental fluorosis, for example, overuse can affect children’s bone development. Sugar substitutes can exert anti-caries effects but large amounts of them may be required to achieve clinical efficacy [10, 13]. Several natural products and their derivatives show potential against *S. mutans*, such as green tea [14], citrus lemon oil [15], and *Galla chinensis* [16].

One of the natural products with the proven anti-caries efficacy is farnesol (3,7,11-trimethyl-2,6,10-dodecatrien-1-ol) in propolis and citrus fruit essential oil, et al. Farnesol increases the permeability of bacterial membranes to protons and reduces glycolytic activity of *S. mutans* in biofilms [17, 18], it inhibits the growth of *Staphylococcus aureus* [19] and *Staphylococcus epidermidis* [20], and also inhibits *Candida albicans* biofilm formation [21]. Our preliminary studies have shown that farnesal (Far; 3,7,11-trimethyl-2,6,10-dodecatrienal), the derivative of farnesol, could inhibit *S. mutans* growth, but it is quite hydrophobic. In the present work, we wished to design a delivery system for Far that would compensate for these disadvantages.

We solubilized Far by linking it to hydrophilic PEG and made this linkage acid-sensitive by conjugating Far to the hydrazine groups of PEG via hydrazone bonds. In this way, we aimed to make Far more selective because it would be released selectively in acidic, cariogenic microenvironments in the oral cavity. We added Tris(tetra-*n*-butylammonium) hydrogen pyrophosphate (TBAP) to the PEG-hyd-Far amphiphilic conjugate, since the pyrophosphate (PPi) moiety binds rapidly to hydroxyapatite [Ca_10_(PO_4_)_6_(OH)_2_] [22, 23], in which hydroxyapatite accounts for 95–96% of the enamel mass [24, 25]. Finally, we inserted the entire conjugate into polymeric micelles (PMs) to further solubilize Far and enhance its bioavailability. Results from in vitro and in vivo rat model of the induced caries suggested that this drug-delivery platform could substantially improve the anti-caries efficacy of Far.

## Results

### Synthesis of pH-sensitive dentotropic PPi-PEG-hyd-Far polymeric conjugate

**Fig. 1:**
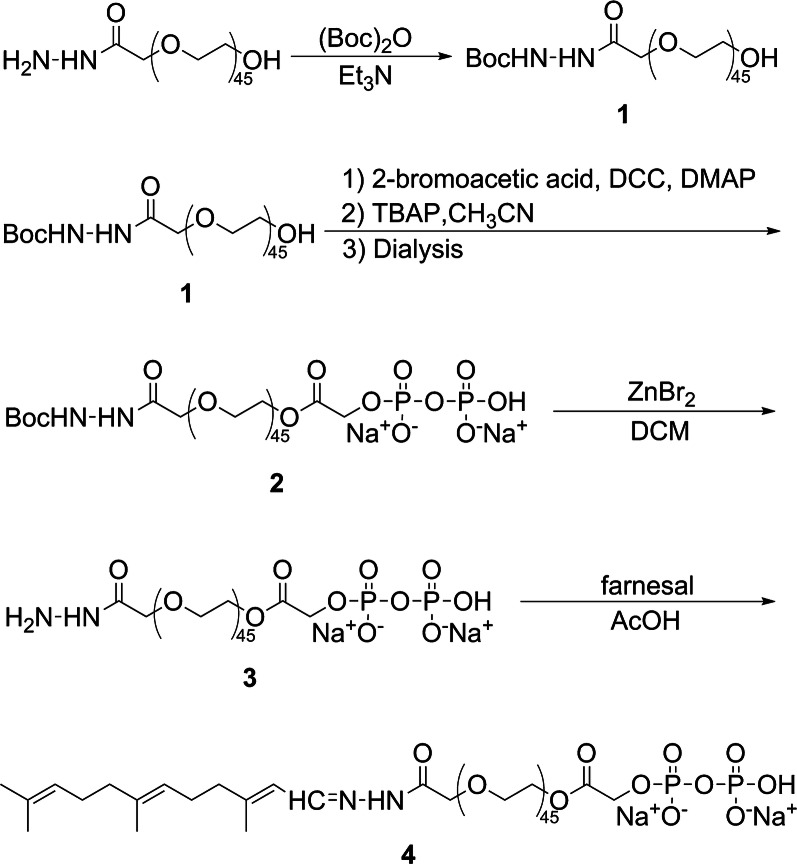
Synthesis of the pH-sensitive dentotropic polymeric conjugate of PPi-PEG-hyd-Far

To develop a pH-sensitive dentotropic polymeric conjugate for the formulations of polymeric micelles, we synthesized the PPi-PEG-hyd-Far according to the synthetic scheme in Fig. 1. The following results from ^1^H and ^13^C NMR confirmed the synthesis of PPi-PEG-hyd-Far as described below.

Compound **1** (Additional file 1: Figure S1) gave a ^1^H NMR spectrum (400 MHz, Chloroform-d) showing δ (ppm) 7.01 (s, 1H), 6.67 (s, 1H), 4.27 (s, 2H), 3.64 (s, 180H), 1.45 (s, 9H). The ^13^C NMR (101 MHz, Chloroform-d) showed δ (ppm) 70.71, 28.40. Compound **2** (Additional file 1: Figure S2) gave a ^1^H NMR spectrum (400 MHz, Chloroform-d) showing δ (ppm) 4.30–4.19 (m, 4H), 3.87 (s, 1H), 3.62 (s, 180H), 2.45 (s, 2H), 1.44 (s, 9H). The ^13^C NMR spectrum (101 MHz, Chloroform-d) showed δ (ppm) 155.82, 70.67, 69.36, 68.88, 65.47, 64.93, 28.37. The ^31^P NMR spectrum (D_2_O, 160 MHz) showed δ (ppm) -9.49 (m, 2P). Compound **3** (Additional file 1: Figure S3) gave a ^1^H NMR spectrum (400 MHz, Chloroform-d) showing δ (ppm) 6.27 (s, 1H), 4.27–4.21 (m, 4H), 3.65 (s, 180H), 1.87 (s, 3H). The ^13^C NMR spectrum (101 MHz, Chloroform-d) showed δ (ppm) 70.72, 69.60, 64.71. Compound **4** (Additional file 1: Figure S4) gave an ^1^H NMR spectrum (400 MHz, Chloroform-d) showing δ (ppm) 8.63 (s, 1H), 7.80 (m, 1H), 6.08 (s, 1H), 5.07 (m, 2H), 4.33 (s, 3H), 3.63 (s, 182H), 2.21 (s, 2H), 2.12 (s, 3H), 2.03 (s, 2H), 1.96 (s, 2H), 1.85–1.82 (m, 2H), 1.66 (s, 3H), 1.57 (s, 6H). The ^13^C NMR spectrum (101 MHz, Chloroform-d) showed δ (ppm) 136.05, 131.67, 124.40, 124.29, 123.32, 123.13, 72.80, 70.70, 70.40, 69.48, 40.20, 39.84, 26.84, 26.21, 25.90, 17.89, 17.47, 16.20.

### Characteristics of polymeric micelles

**Fig. 2:**
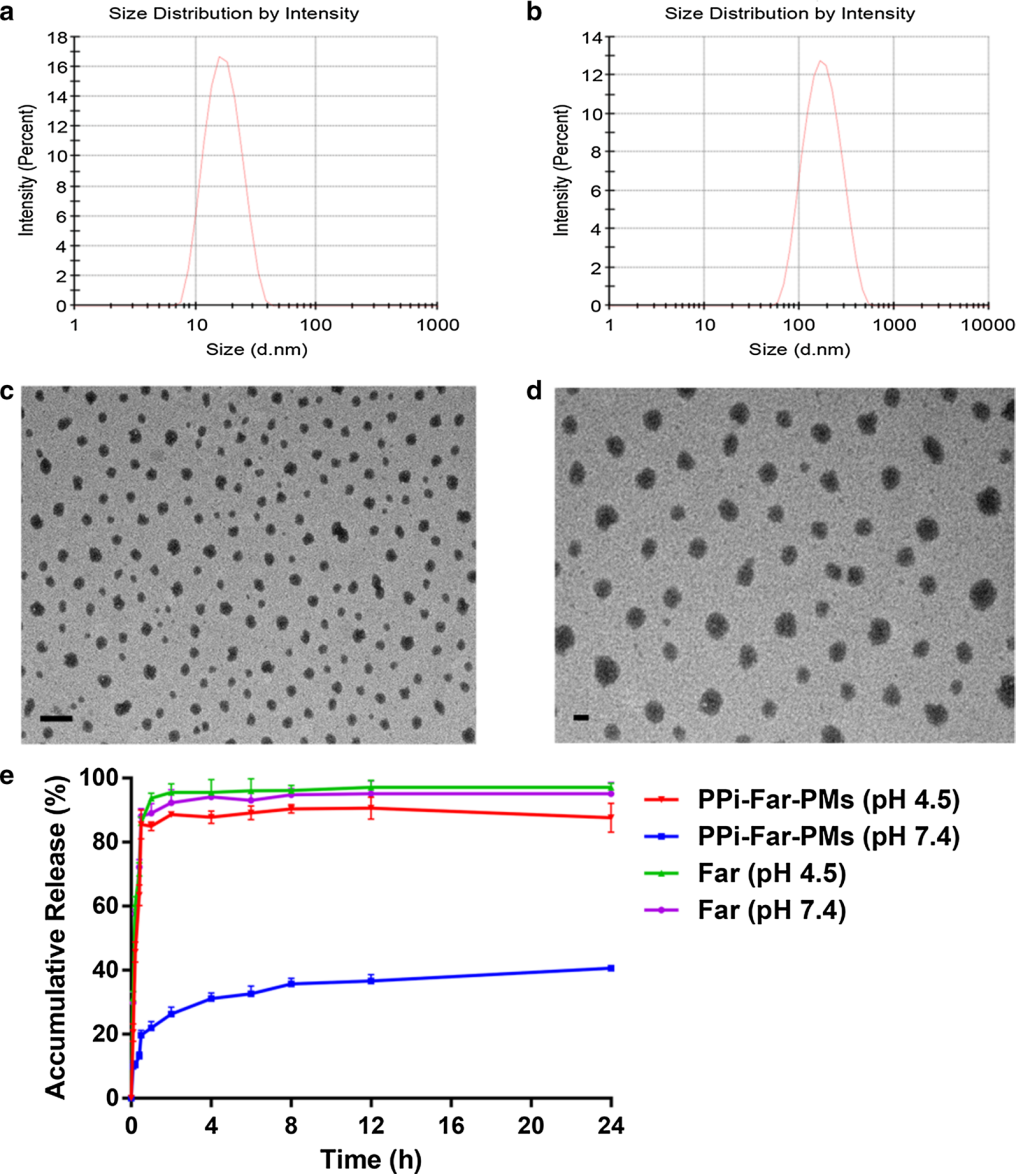
*In vitro* characterization of Blank PMs and PPi-Far-PMs. Particle size distribution of Blank PMs (**a**) and PPi-Far-PMs (**b**) was measured using a particle size analyzer. Morphology of Blank PMs (**c**) and PPi-Far-PMs (**d**) were observed by transmission electron microscopy. The accumulative release (%) of farnesal was assessed in phosphate-buffered saline at pH 4.5 and 7.4 at 37 °C (**e**). Results are presented as mean ± SD (n = 3). The bar in both **c** and **d** is 100 nm

The pH-sensitive dentotropic PPi-PEG-hyd-Far and mPEG_2000_-PLA_2000_ polymeric conjugate were self-assembled into PPi-Far-PMs using a film hydration procedure. Dynamic light scattering showed the resulting blank PMs to have an average diameter of 15.95 ± 0.10 nm with a polydispersity index of 0.086 ± 0.008 (Fig. 2a) and zeta potential of − 0.71 ± 0.07 mV. PPi-Far-PMs were much larger, with a diameter of 146.20 ± 0.87 nm, polydispersity index of 0.234 ± 0.012 (Fig. 2b) and zeta potential of − 4.92 ± 0.24 mV. Far-PMs and PPi-Far-PMs were similar in size and zeta potential. In all preparations, the polydispersity index was less than 0.3, suggesting particle uniformity. Consistent with this idea, transmission electron microscopy showed PMs and PPi-Far-PMs to be uniformly spherical (Fig. 2c, d). The CMCs of blank PMs and PPi-Far-PMs were, respectively, 2.76 × 10^−3^ and 7.92 × 10^−4^ mg/mL (Additional file 1: Figure S5A, B).

We developed an HPLC method to calculate drug loading and encapsulation efficiencies of the PMs. The retention time of Far in PMs was consistent with that of the Far standard, and no interference signals were observed near the characteristic peak. The Far standard curve was linear over the range of 10–400 μg/mL (Abs = 15,146 * Conc—29,860, R^2^ = 0.9992). Intra- and inter-day relative standard deviation (RSD) was less than 10%, and Far extraction recoveries were 99.09 ± 0.48% (RSD, 0.48%) at low concentrations, 99.84 ± 0.10% (RSD, 0.10%) at medium concentrations, and 101.63 ± 0.04% (RSD, 0.04%) at high concentrations. These results suggest that our HPLC method was robust and reliable. Using this method, we measured drug loading and encapsulation efficiencies to be 9.10 ± 0.70% and 76.4 ± 2.10% for Far-PMs, and 9.51 ± 0.40% and 78.30 ± 1.40% for PPi-Far-PMs.

Far was released from PPi-Far-PMs much faster at pH 4.5 than at pH 7.4 (Fig. 2e). At pH 4.5, nearly 90% of Far was released within the first 30 min, compared to only 40.60% of Far released within 24 h at pH 7.4, that may depends on the pH sensitivity of the hydrazone bond. These results suggest that we succeeded in creating a Far delivery system that selectively and rapidly released the drug under acidic conditions.

### PPi-Far-PMs bind to hydroxyapatite particles better and faster than Far-PMs

**Fig. 3:**
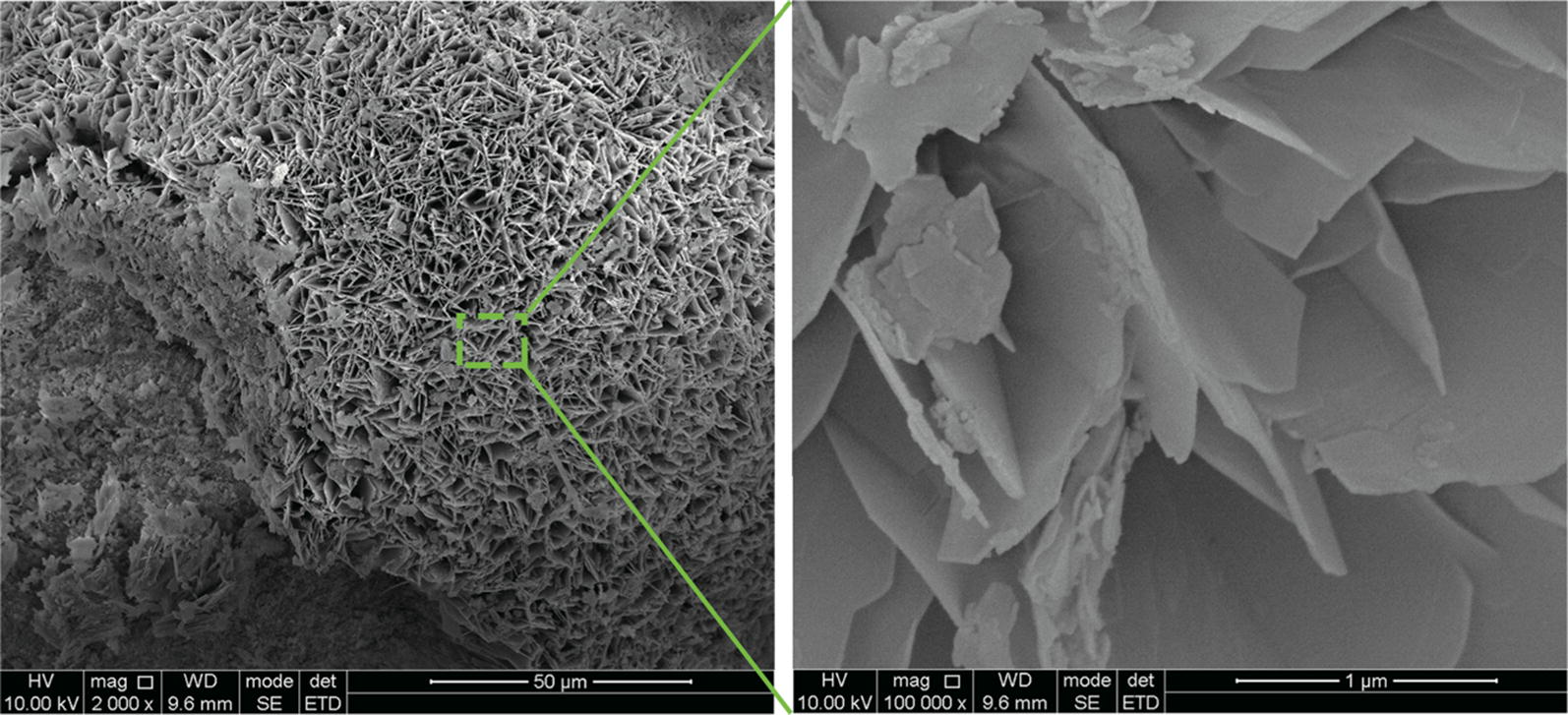
Preparation of biotechnological hydroxyapatite. During preparation, the top surface of the small intestinal submucosa membrane was maintained in contact with K_2_HPO_4_ solution, while the lower surface was kept in contact with the Ca(CH_3_COO)_2_ solution. After incubation for 10 days at 37 °C, the entire submucosa membrane with hydroxyapatite was peeled off and analyzed by scanning electron microscopy. The magnification in the left image is ×2000, and the right image shows a zoomed view at ×100000

**Fig. 4:**
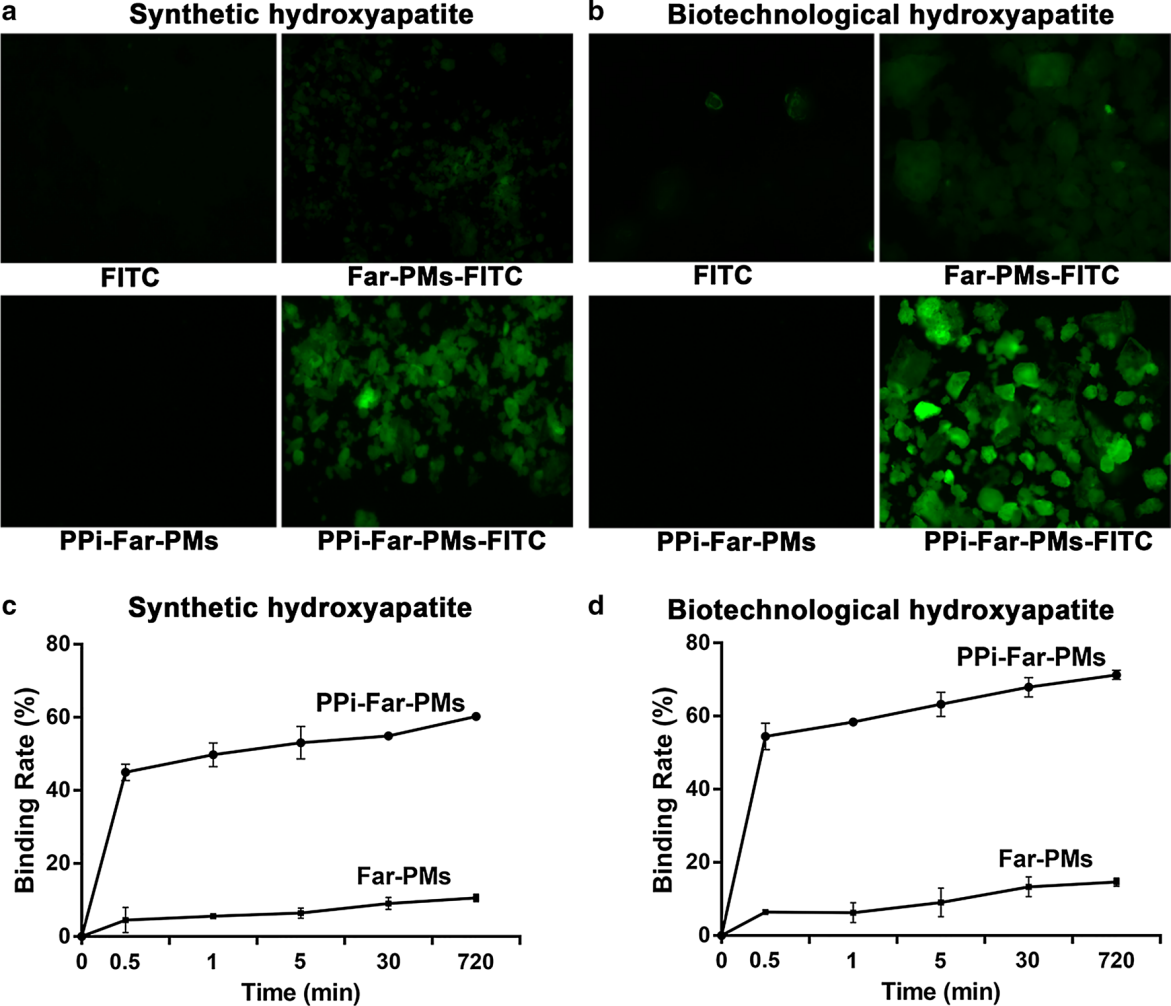
The binding ability of PPi-Far-PMs to hydroxyapatite particles. PPi-Far-PMs and Far-PMs were labeled with FITC and incubated with the commercial synthetic hydroxyapatite (**a**, **c**) or with the biotechnological hydroxyapatite (**b**, **d**). The images show 30-min incubations with FITC, Far-PMs-FITC, PPi-Far-PMs or PPi-Far-PMs-FITC. Magnification, ×40. Binding kinetics (**c**, **d**) were assessed by assaying the bound Far using HPLC at different time points. All data are mean ± SD (n = 3)

We prepared the biotechnological hydroxyapatite (Fig. 3) as a mimic of enamel in order to examine the binding of PPi-Far-PMs. PPi-Far-PMs showed much greater ability to bind hydroxyapatite than Far-PMs (Fig. 4a, b). PPi-Far-PMs bound efficiently to hydroxyapatite particles within 2 min (Fig. 4c, d), which is comparable to the time most people spend brushing their teeth or rinsing their mouth with mouthwash. PPi-Far-PMs also showed greater binding rate with biotechnological hydroxyapatite than that with the commercial hydroxyapatite at each time point.

### Anti-bacterial effects of Far against *S. mutans*

Since *S. mutans* is the main microorganism initiating caries, the MIC and MBC of Far against this microbe were measured. The bacteria appeared to be similarly susceptible to Far and farnesol based on MIC (14 *vs* 28 μg/mL) and MBC (112 *vs* 112 μg/mL; Additional file 1: Table S1) [26]. The positive control treatment with CHX showed lower MIC and MBC, but it was associated with tooth staining and calculus formation as noted previously [10].

### PPi-Far-PMs enhance the anti-caries activity of Far in vivo

**Fig. 5:**
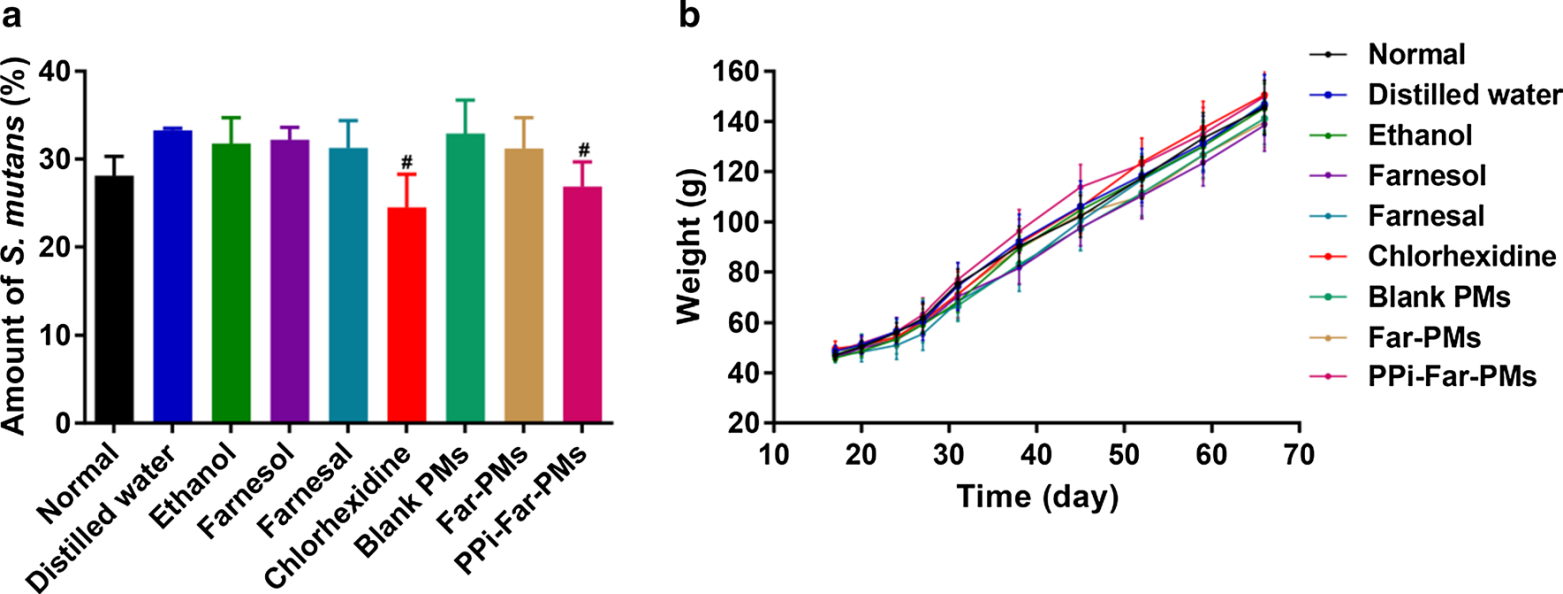
Measurement of the relative levels of *S. mutans* (**a**) and body weight of rats (**b**) in the dental caries model after treatment with distilled water, 15% ethanol, farnesol, farnesal, chlorhexidine, blank PMs, Far-PMs and PPi-Far-PMs, respectively. Values are expressed as mean ± standard deviation. ^#^*P *< 0.05 *vs* the control group treated with distilled water

Blank PMs and distilled water had no obvious effect on the amount of *S. mutans* in saliva from the treated rats (Fig. 5a). The other all treatments reduced the amount of *S. mutans*, while CHX and PPi-Far-PMs reduced it (*P *< 0.05) significantly. These results indicate that PPi-Far-PMs can inhibit the growth of *S. mutans* in vivo. All animals remained in good health during the experiment and the body-weight increase was similar to that of the normal rats rats (Fig. 5b). We observed that the physiological conditions of rats such as diet and defecation are normal. No local oral mucosal allergy was observed in any of the animals during the drug intervention.

**Fig. 6:**
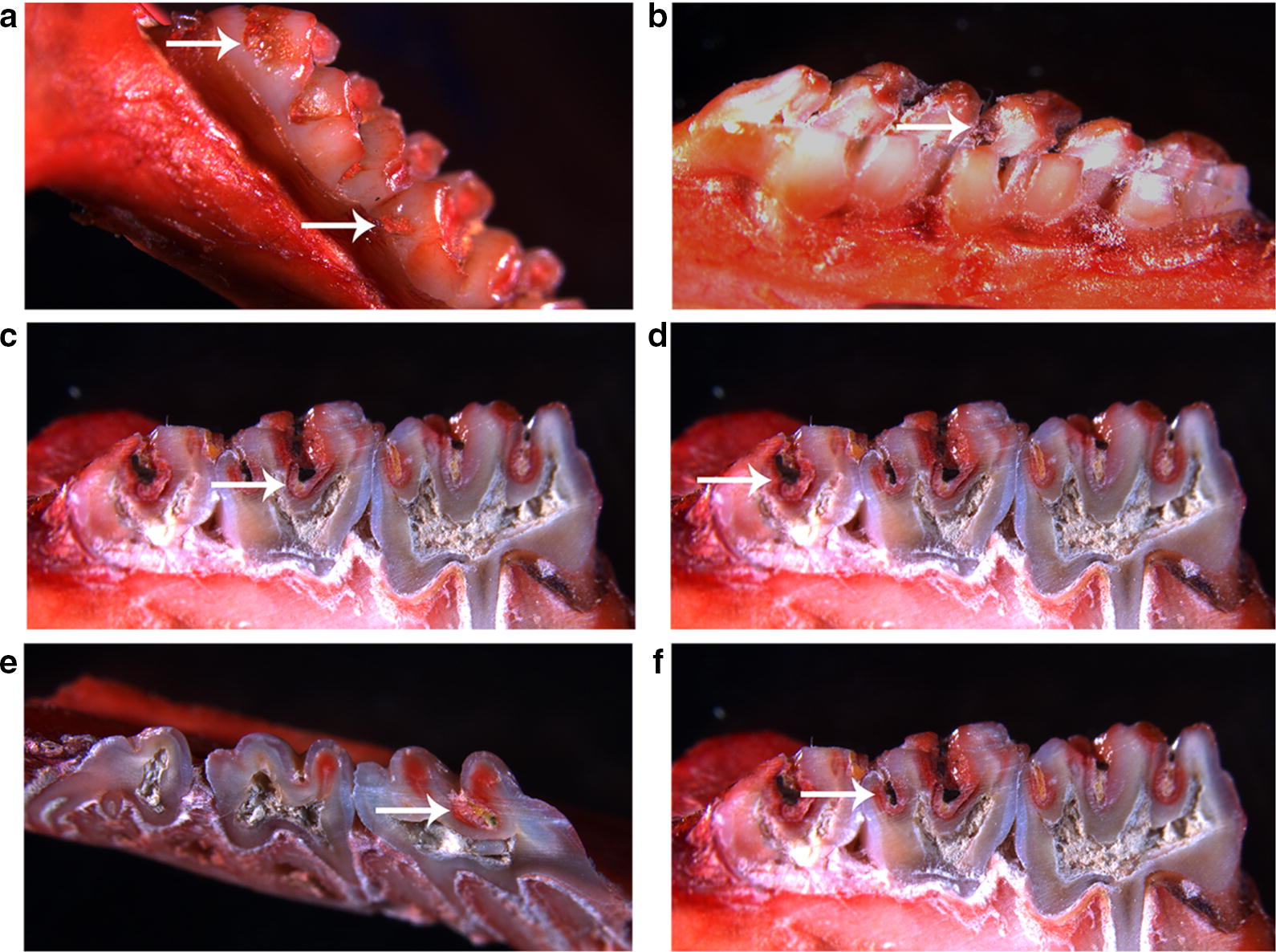
Representative stereo-micrographs of molars from the rat model of caries, showing smooth-surface caries with enamel affected (**a**), proximal-surface with enamel affected (**b**), sulcal-surface with enamel affected (**c**), sulcal-surface with dentin exposed (**d**), sulcal-surface with 3/4 of the dentin affected (**e**), and sulcal-surface with whole dentin affected (**f**). Bold arrows show examples of the indicated damage. Magnification, 14×

**Fig. 7:**
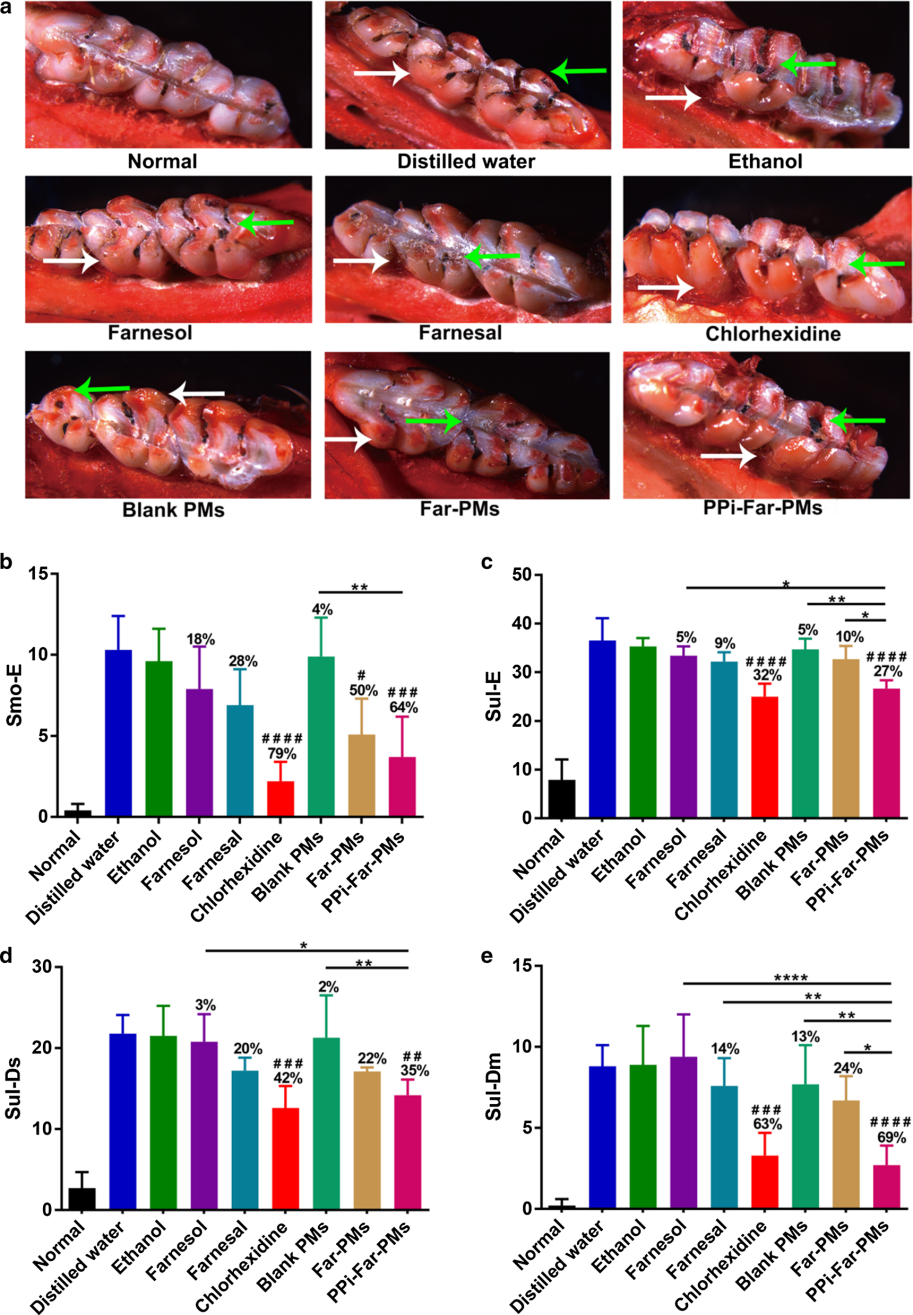
Treatments effects on the development of dental caries in rats. Animals were treated with distilled water, 15% ethanol, farnesol, farnesal, chlorhexidine, blank PMs, Far-PMs and PPi-Far-PMs, respectively. **a** The smooth-surface and sulcal-surface caries of molars were observed under a stereomicroscope (×14). The white arrow indicated the smooth-surface caries while the green arrow indicating the sulcal-surface caries. Quantitative assessment was based on carious lesion severity of smooth-surface and sulcal-surface according to the Keyes’ scoring system, including **b** the smooth-surface with enamel affected (Smo-E), **c** sulcal-surface with enamel affected (Sul-E), **d** sulcal-surface with dentin exposed (Sul-Ds), **e** sulcal-surface with 3/4 of the dentin affected (Sul-Dm). Values are expressed as mean ± standard deviation (n = 7). The Tamhane’s T2 test (*P < 0.05, **P < 0.01, ****P < 0.0001) was used to assess the treatment efficacy. Symbols represented statistical significance of the labeled groups relative to the group treated with distilled water (^#^*P *< 0.05, ^##^*P *< 0.01, ^###^*P *< 0.001, ^####^*P *< 0.0001). The caries reduction rate was determined as described in Methods

Figure 6 shows examples of treated rats that showed Smo-E, Pro-E, Sul-E, Sul-Ds, Sul-Dm or Sul-Dx. The treatments differed in how much and how severely they caused sulcal-surface caries of molars (Fig. 7a); as expected, distilled water was associated with the most severe caries. Keyes’ scores of smooth-surface caries were significantly higher with distilled water than with Far-PMs (*P* < 0.05), PPi-Far-PMs *(P* < 0.001), or CHX (*P* < 0.0001) (Table 1 and Fig. 7b). Farnesol reduced incidence of Smo-E by 18%; Far, by 28%; and ethanol, by 15%. In contrast, Far-PMs, PPi-Far-PMs and CHX reduced incidence by, respectively, 50%, 64% and 79%.

**Table 1:** Keyes’ scores for assessment the efficiency of different treatments on the development of dental caries in rats

Treatment	Smooth-surface caries	Sulcal-surface caries			
	E	E	Ds	Dm	Dx
Distilled water	10.3 (2.1)^a^	36.5 (4.6)^a^	21.8 (2.3)^a^	8.8 (1.3)^a^	0.7 (0.8)^a^
15% Ethanol	9.6 (2.0)^a^	35.3 (1.7)^ac^	21.5 (3.7)^a^	8.9 (2.4)^a^	0.5 (0.5)^a^
Farnesol	7.9 (2.6)^ac^	33.4 (1.9)^ac^	20.8 (3.4)^a^	9.4 (2.6)^a^	0.5 (0.7)^a^
Farnesal	6.9 (2.2)^ac^	32.2 (1.9)^acd^	17.2 (1.6)^ac^	7.6 (1.7)^a^	n.d.
Chlorhexidine	2.2 (1.2)^bd^	25 (2.7)^b^	12.6 (2.7)^bc^	3.3 (1.4)^bcde^	n.d.
Blank PMs	9.9 (2.4)^a^	34.7 (2.2)^ac^	21.3 (5.2)^a^	7.7 (2.4)^a^	0.4 (0.4)^a^
Far-PMs	5.1 (2.2)^bc^	32.7 (2.7)^ac^	17.1 (0.5)^ac^	6.7 (1.5)^ac^	n.d.
PPi-Far-PMs	3.7 (2.5)^bcd^	26.7 (1.7)^bd^	14.2 (1.9)^bd^	2.7 (1.2)^bde^	n.d.

The Keyes’ scores are presented as the average of all the measurements (n = 7). Values followed by the same superscripts (a, b, c, d, e) are not significantly different from each other (*P *>0.05; comparison for all pairs using Tamhane’s T2 test)

E, enamel affected; Ds, dentin exposed; Dm, 3/4 of the dentin affected; and Dx, whole dentin affected; n.d., not detectable

The incidence of Sul-E was significantly higher with distilled water than with PPi-Far-PMs (27% lower, *P* < 0.0001), or CHX (32% lower, *P* < 0.0001) (Fig. 7c). In contrast, the incidence of Sul-E was only 10% lower with Far-PMs, 9% lower with Far and 5% lower with farnesol. Incidence of Sul-Ds was 35% lower with PPi-Far-PMs and 42% lower with CHX than the control group treated with distilled water (Fig. 7d). The corresponding reductions in Sul-Dm incidence were 69% and 63% (Fig. 7e). These results suggest that Far-loaded pH-sensitive dentotropic PMs significantly improve the anti-caries effect of free Far. PPi-Far-PMs and CHX inhibit sulcal-surface caries to a similar extent.

### PPi-Far-PMs enhance the teeth mechanical strength in rat caries

**Fig. 8:**
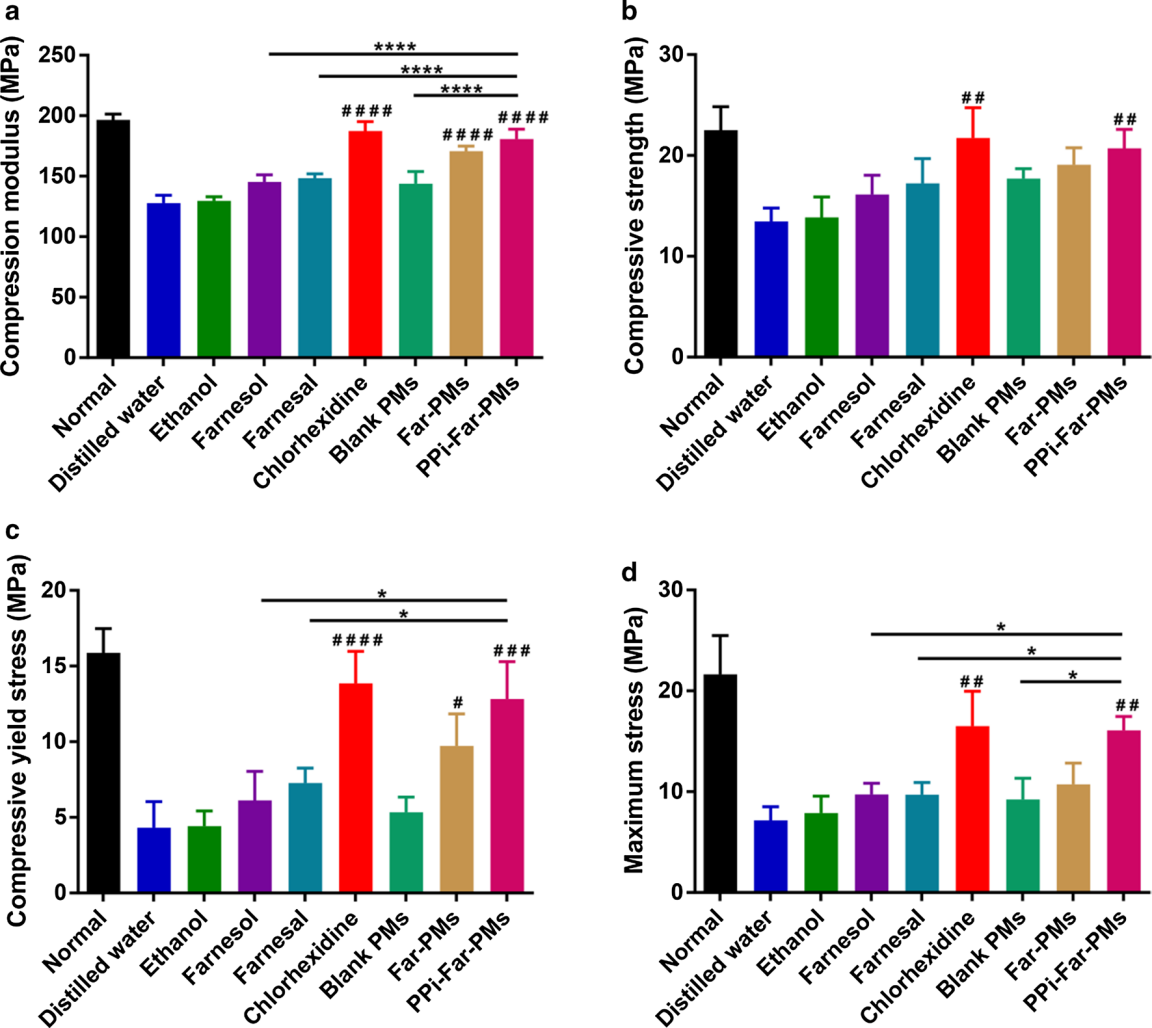
Effects of different treatments on the biomechanical properties of molars including the compression modulus (**a**), compressive strength (**b**), compressive yield stress (**c**) and maximum stress (**d**). Rats were treated with distilled water, 15% ethanol, farnesol, farnesal, chlorhexidine, blank PMs, Far-PMs and PPi-Far-PMs, respectively. At 5 weeks after administration, the molars were subjected to the mechanical compressive strength testing via a biomechanical testing system. Values are expressed as mean ± SD. The Tamhane’s T2 test test (*P < 0.05, ****P < 0.0001) was used to assess the treatment efficacy. Symbols represented statistical significance of the labeled groups with the group treated with distilled water (^#^*P *< 0.05, ^##^*P *< 0.01, ^###^*P *< 0.001, ^####^*P *< 0.0001)

Molars mechanical strength was much higher in teeth from rats treated with PPi-Far-PMs than that in teeth from rats treated with Far, which in turn was higher than in teeth from animals treated with distilled water (Fig. 8a, d). PPi-Far-PMs exerted similar effects as CHX under these conditions.

### PPi-Far-PMs can restore the microarchitecture of teeth with caries

**Fig. 9:**
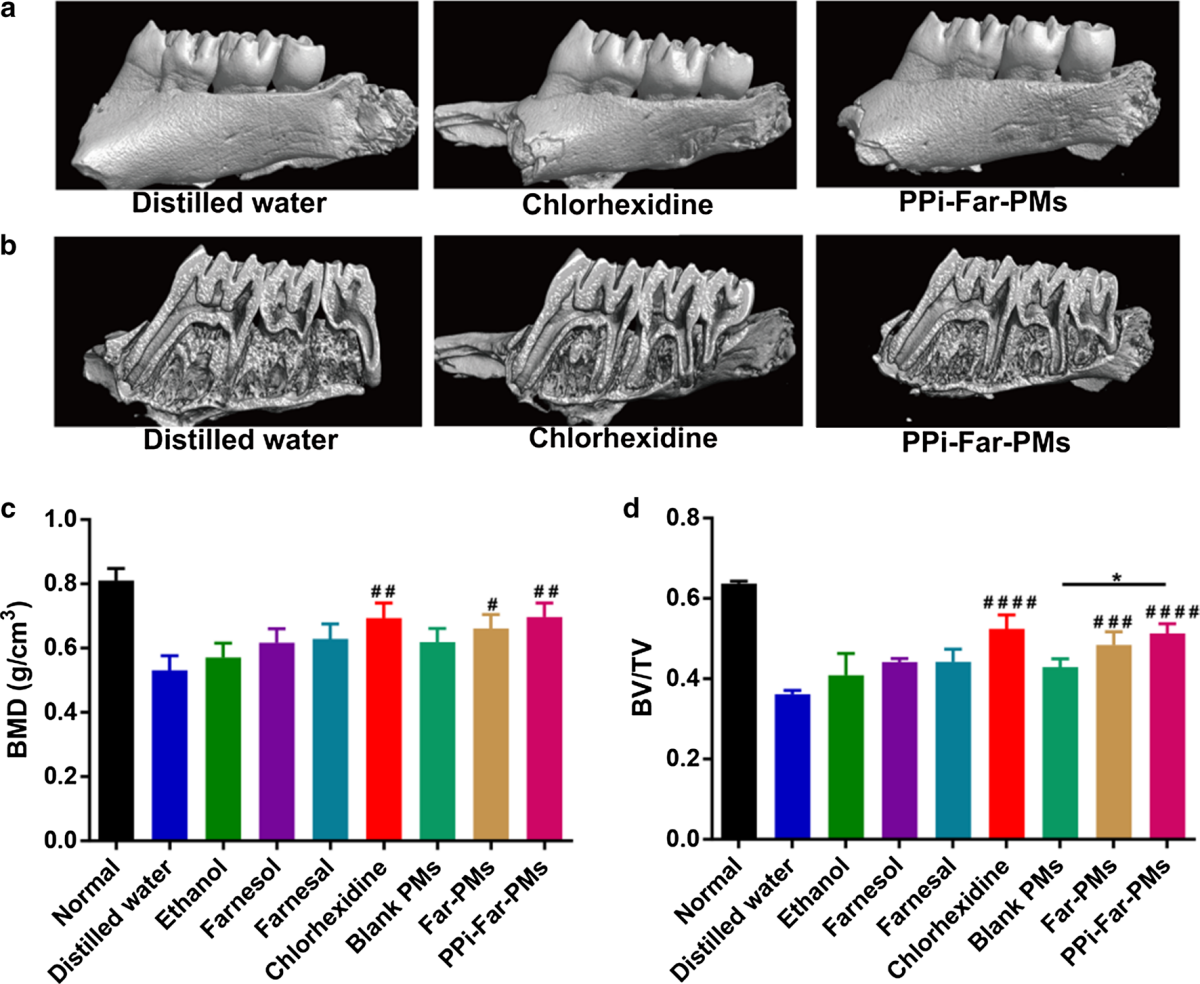
Effects of different treatments on the microarchitecture of molars in rats. Animals were treated with distilled water, 15% ethanol, farnesol, farnesal, chlorhexidine, blank PMs, Far-PMs and PPi-Far-PMs, respectively. After 5 weeks, the molars of rats were analyzed using high-resolution micro-computed tomography to obtain three-dimensional reconstruction pictures of the smooth-surface molars (**a**) and sulcal-surface molars (**b**). The bone mineral density (BMD, **c**) and bone volume per tissue volume (BV/TV, D) were quantitatively measured. Data were expressed as mean ± SD (n = 7). The Tamhane’s T2 test (*P < 0.05) was used to assess for treatment efficacy. Symbols represented statistical significance of the labeled groups with the group treated with distilled water (^#^*P *< 0.05, ^##^*P *< 0.01, ^###^*P *< 0.001, ^####^*P *< 0.0001)

The caries caused severe molars damage, as seen in BMD and BV/TV of molars from animals treated with distilled water or blank PMs. BMD and BV/TV of molars were significantly higher in groups of Far-PMs and PPi-Far-PMs than that in group of the distilled water (Fig. 9, Additional file 1: Figure S6). The improvement in the group of PPi-Far-PMs was similar to that in the group of CHX. None of the other treatments significantly improved BMD or BV/TV relative to the distilled water. These results indicate that PPi-Far-PMs can effectively inhibit demineralization of the tooth enamel.

## Discussion

We have synthesized pH-sensitive dentotropic PPi-PEG-hyd-Far polymeric conjugate by linking PEG and Far via an acid-labile hydrazone bond, then modifying it with biodegradable PPi to make enamel-targeting PMs. PEG is a non-immunogenic, biocompatible water-soluble polymer often conjugated to therapeutic proteins and other drugs. Several of these conjugates have entered clinical trials for cancer treatment [27–30]. The hydrazone linkage has often been used to render the release of drugs such as doxorubicin and paclitaxel selective under acidic conditions [31–34]. We prepared PPi-Far-PMs using a simple membrane hydration method that generated homogeneous, uniformly spherical micelles.

We found that Far, a derivative of the natural product farnesol, shows similar anti-bacterial efficacy as farnesol against *S. mutans*. At the same time, its incorporation into PPi-Far-PMs helps its retention in the oral cavity and led to stronger anti-bacterial and anti-caries effects than free farnesol or even Far-PMs in our rat model of induced caries. While *S. mutans* is a major component of dental plaque that causes cavities, other bacterial species also contribute, including *Streptococcus sobrinus*, *lactobacilli* and *candida albicans*. Future work should examine the efficacy of Far against these species in order to gain a more complete understanding of its anti-caries activity.

Consistent with the good anti-caries activity of PPi-Far-PMs in our rat model, we found that the micelles bound strongly to biotechnological hydroxyapatite particles; in fact, they bound more strongly to these particles, which mimic tooth material, than to chemical hydroxyapatite. PPi-Far-PMs bound within a few minutes to hydroxyapatite and persisted for up to 12 h, which suggests they can be effective during routine use.

We assayed the anti-caries activity of PPi-Far-PMs in a rat model of caries induced by the combination of *S. mutans* UA159 infection and a cariogenic diet [35, 36]. PPi-Far-PMs significantly reduced the incidence and severity of smooth and sulcal surface caries compared with the control group, while free Far and farnesol showed no obvious difference from controls. Mechanical tests and micro-CT of molars showed that PPi-Far-PMs effectively protected teeth from damage and bone loss. Meanwhile, we also found that when the PPi-Far-PMs drug-loading concentration was at low-dose of 0.55 mg/mL, Far can reduce the incidence of Smo-E by 51%, and that of Sul-E by 17%, respectively. In compressive strength experiments, resulted from the low-dose group of PPi-Far-PMs, values of compressive strength, compressive strength, compressive yield stress and maximum stress were 172.49 ± 5.89 MPa, 20.50 ± 2.33 MPa, 10.78 ± 1.65 MPa, and 15.47 ± 1.90 MPa, respectively; while in distilled water group, they were 127.77 ± 6.45 MPa, 13.47 ± 1.32 MPa, 4.32 ± 1.72 MPa, and 7.17 ± 1.34 MPa, respectively. Statistical comparisons indicated that the low-dose of PPi-Far-PMs treatments showed remarkable differences compared to distilled water (*P* < 0.0001, *P* < 0.05, *P* < 0.01, *P* < 0.01). Meanwhile, we found that BMD and BV/TV of molars were significantly higher with the low-dose PPi-Far-PMs (BMD 0.694 ± 0.041 g/cm^3^, BV/TV 0.505 ± 0.005) than with distilled water (BMD 0.531 ± 0.045 g/cm^3^, BV/TV 0.362 ± 0.010; *P *< 0.01, *P *< 0.0001). This reminds us that the drug-delivery system of PPi-Far-PMs can reduce the dosage of the Far and prevent effectively the dental caries. We attribute these results to the ability of PPi-Far-PMs killing bacteria in the acidic microenvironment of dental plaques and binding efficiently to hydroxyapatite, and thereby inhibited demineralization and promoted re-mineralization.

## Conclusions

In this study, we developed the drug-delivery system of PPi-Far-PMs that binds efficiently and rapidly to hydroxyapatite, and that releases Far selectively in the acidic microenvironment of dental plaque. We showed in vitro that Far on its own has similar anti-bacterial activity as its parent farnesol against cariogenic *S. mutans* UA159. We further showed that incorporating Far into PPi-Far-PMs made it much more effective than free farnesol at treating dental caries in a rat model. This suggests that PMs can improve the water solubility and prolong the retention time in the oral cavity. This novel pH-sensitive drug delivery system shows potential for targeted anti-bacterial treatment against dental caries, and it may be useful for delivering other agents to treat disease in the oral cavity.

## Materials and methods

### Materials

Far, farnesol, and TBAP were supplied by Sigma-Aldrich (St. Louis, MO, USA). Chlorhexidine digluconate (CHX) was obtained from Macklin (Shanghai, China). HO-PEG_2000_-NHNH_2_ was obtained from Xi’an Ruixi Biological Technology (Xi’an, China), and mPEG_2000_-PLA_2000_ was purchased from Jinan Daigang Biomaterials (Jinan, China). Mitis Salivarius agar was purchased from BD Biosciences (San Jose, CA, USA). Brain heart infusion broth and agar were purchased from Hopebio (Qingdao, China). *S. mutans* UA159 (ATCC 700610) was purchased from the China Center of Industrial Culture Collection (Beijing, China).

### Synthesis of pH-sensitive dentotropic polymeric conjugate

The pH-sensitive dentotropic polymeric conjugate PPi-PEG-hyd-Far was synthesized as shown in Fig. 1. The commercially available HO-PEG_2000_-NHNH_2_ was protected with a *t*-butyloxy carbonyl (Boc) group, and then subjected to esterification and bromination to give PPi-PEG-NHNH-Boc. The Boc group was removed and subsequent hydrazide reaction generated PPi-PEG-hyd-Far, whose identity was confirmed using ^1^H and ^13^C NMR. The specific reaction conditions for each compound are detailed below.

### Synthesis of compound 1: tert-butyl 2-(2-(2-hydroxyethoxy)acetyl)hydrazine-1-carboxylate (HO-PEG-NHNH-Boc)

Triethylamine (25.3 mg, 0.25 mmol) was added to a stirred solution of HO-PEG_2000_-NHNH_2_ (500 mg, 0.25 mmol) in methanol. The mixture was cooled to 0 °C and di-*tert*-butyl dicarbonate (65.5 mg, 0.30 mmol) was added slowly to the reaction solution. The reaction mixture was stirred at 0 °C for 1 h, then warmed to room temperature and stirred for 24 h. The solution was concentrated under vacuum, and the product was precipitated in anhydrous ether three times, dried overnight under vacuum and stored at − 20 °C for an overall yield of 72.4%.

### Synthesis of compound 2: sodium 2,2-dimethyl-4,7,13-trioxo-3,9,12-trioxa-5,6-diazatetradecan-14-yl hydrogen diphosphate (PPi-PEG-NHNH-Boc)

Bromoacetic acid (43.4 mg, 0.31 mmol) was added to a stirred solution of compound **1** (500 mg, 0.24 mmol) in anhydrous dichloromethane (DCM). The solution was cooled to 0 °C, then 4-dimethylaminopyridine (DMAP, 2.9 mg, 0.024 mmol) and N,N′-dicyclohexylcarbodiimide (DCC, 54.4 mg, 0.26 mmol) were added slowly, and the mixture was stirred at room temperature for 24 h. The reaction mixture was filtered and concentrated under vacuum, and the product was precipitated in anhydrous ether. The precipitate was filtered and dialyzed against water for 24 h. TBAP (415.1 mg, 0.46 mmol), previously dissolved in anhydrous acetonitrile (CH_3_CN), was added slowly to the dialyzed product, and the mixture was stirred at room temperature for 12 h. The solution was concentrated under vacuum, and the product was precipitated into anhydrous ether. The product was filtered, dialyzed against NaCl solution and then dialyzed against water at 0 °C for 10 h. The purified product was freeze–dried and stored at − 20 °C for overall yield of 80.7%.

### Synthesis of compound 3: sodium 2-(2-(2-hydrazinyl-2-oxoethoxy)ethoxy)-2-oxoethyl hydrogen diphosphate (PPi-PEG-NHNH_2_)

Compound **2** (500 mg, 0.18 mmol) was de-protected using zinc bromide (ZnBr_2_, 81 mg, 0.36 mmol) in dichloromethane (DCM) for 4 h. The solution was filtered and concentrated under vacuum, and the product was precipitated in anhydrous ether three times and against water at 0 °C for 10 h. Then the purified product was dried overnight under vacuum and stored at − 20 °C with overall yield of 80.20%.

### Synthesis of compound 4: sodium 2-oxo-2-(2-(2-oxo-2-(2-((2E,6E)-3,7,11-trimethyldodeca-2,6,10-trien-1-ylidene)hydrazinyl)ethoxy)ethoxy)ethyl hydrogen diphosphate (PPi-PEG-hyd-Far)

Compound **3** (500 mg, 0.19 mmol) and Far (61.0 mg, 0.28 mmol) were dissolved in anhydrous methanol, and acetic acid (1.1 mg, 0.02 mmol) was added to the reaction solution. After stirring at room temperature for 48 h, the solution was concentrated under vacuum, and the product was precipitated in anhydrous ether three times. Then the purified product was dried overnight under vacuum and stored at − 20 °C with overall yield of 79.80%.

### Preparation of polymeric micelles

The membrane hydration method [37] was used to prepare blank polymeric micelles (PMs), Far-loaded pH-sensitive polymeric micelles (Far-PMs), as well as PPi-targeted and Far-loaded pH-sensitive dentotropic polymeric micelles (PPi-Far-PMs). The following components were used: mPEG_2000_-PLA_2000_ (A), PEG-hyd-Far (B), PPi-PEG-hyd-Far (C), and Far (D). The weight ratios of components A/B/C/D were optimized using the central composite design [38, 39] to be as following (Table 2): blank PMs, 40/0/0/0; Far-PMs, 20/20/0/4; and PPi-Far-PMs, 20/0/20/4. The components were dissolved in 4.0 mL of acetonitrile in a round-bottom flask, then the acetonitrile was evaporated under vacuum at 55 °C to obtain a thin film. Residual acetonitrile was completely removed under vacuum overnight at room temperature. The dried thin film was hydrated with ultra-pure water (2.0 mL) and sonicated for 3 min in a bath sonicator, stirred for 12 h at room temperature and filtered through a 0.22-µm membrane. The micellar solution was freeze-dried and stored at 4 °C.

**Table 2:** Composition and characterization of polymeric micelles

Polymeric micelles	Components (weight ratio, A/B/C/D)	Particle size (nm)	Polydispersity index	Zeta potential (mV)	Drug loading efficiency	Encapsulation efficiency
Blank PMs	40/0/0/0	15.95 ± 0.10	0.086 ± 0.008	− 0.71 ± 0.07	–	–
Far-PMs	20/20/0/4	136.17 ± 0.49	0.268 ± 0.006	− 2.72 ± 0.14	9.10 ± 0.70%	76.40 ± 2.10%
PPi-Far-PMs	20/0/20/4	146.20 ± 0.87	0.234 ± 0.012	− 4.92 ± 0.24	9.51 ± 0.40%	78.30 ± 1.40%

A, mPEG_2000_-PLA_2000_; B, PEG-hyd-Far; C, PPi-PEG-hyd-Far; D, Farnesal. Values are mean ± SD from three independent experiments

The same method and the additional reagent FITC-PEG (E) were used to prepare FITC-labeled PMs in the following weight ratios: Far-PMs-FITC, A/B/D/E: 20/20/4/1; and PPi-Far-PMs-FITC, A/C/D/E: 20/20/4/1.

### Characterization of polymeric micelles

The size and zeta potential of polymeric micelles were measured using dynamic light scattering (Zetasizer Nano ZS90, Malvern Instruments, Malvern, UK). Morphology of blank PMs and PPi-Far-PMs were observed using transmission electron microscopy (JEM-100SX, Japan). Critical micelle concentration (CMC) was determined using fluorescence spectroscopy and pyrene as the hydrophobic fluorescent probe.

Encapsulation and drug-loading efficiencies were calculated using the ultrafiltration method and an ultrafiltration column with a molecular weight cut-off of 3 kDa (Solarbio Science and Technology, Beijing, China). Far was quantified on an Agilent ZORBAXSB-C18 column (4.6 × 150 mm, 5 µm) at 25 °C attached to an Agilent 1260 HPLC system (Infinity, USA). The mobile phase was acetonitrile and ultrapure water (80:20, v/v) and the flow rate was 1 mL/min. The detection wavelength was 216 nm. Encapsulation efficiency was calculated using the equation:

$$Encapsulation \, efficiency \, \left( \% \right) \, = \, (Weight \, of\, Far \, in \, micelles) / \left( {Weight \, of \, total \, Far} \right) \, \times \, 100\%$$

Drug-loading efficiency was calculated using the equation:

$$Drug - loading \, efficiency \, \left( \% \right) \, = \, {{(Weight \, of\, Far \, in \, micelles) \, } \mathord{\left/ {\vphantom {{(Weight \, of\, Far \, in \, micelles) } {\left( {Weight \, of \, total \, micelles} \right)}}} \right. \kern-0pt} {\left( {Weight \, of \, total \, micelles} \right)}} \, \times \, 100\% .$$

Release of Far from PPi-Far-PMs was investigated in vitro using the dialysis method. Briefly, 1 mL of PPi-Far-PMs or free Far were placed into separate dialysis bags with a molecular weight cut-off of 1 kDa (Spectrumlabs, USA) and dialyzed at 37 °C against phosphate-buffered saline (PBS) at pH 4.5 and 7.4 with gentle stirring. At different time points (0.1, 0.2, 0.4, 0.5, 1, 2, 4, 6, 8, 12 and 24 h), 0.5 mL of release medium was removed and replaced with fresh medium. Far was quantified by HPLC as described above, and experiments were performed three times.

### Binding of PPi-Far-PMs to hydroxyapatite particles in vitro

As we all know, the most important component of the tooth enamel is hydroxyapatite [24]. To mimic the binding process of PPi-Far-PMs with tooth enamel, we used a small intestinal submucosa as the bio-mineralization template to prepare plate-like, single-crystal hydroxyapatite, referred to henceforth as ‘biotechnological hydroxyapatite’. Meanwhile, we used commercially available hydroxyapatite (Macklin, Shanghai, China) as the control.

### Synthesis and characterization of hydroxyapatite

Small intestinal submucosa was prepared as described [40]. The reaction device included a beaker and a centrifuge tube with a hole in the middle of the cap. The submucosa membrane covered the hole to seal the centrifuge tube, which was filled with a solution of K_2_HPO_4_ (30 mL, 0.1 M). This tube was inverted and soaked into the beaker filled with a solution of Ca(CH_3_COO)_2_ (30 mL, 0.1 M). This reaction system, which mimics bone mineralization conditions, was incubated at 37 °C for 10 days. Care was taken to ensure that the top and bottom surfaces of the submucosa membrane remained in contact with liquid [41]. Morphology of the biotechnological hydroxyapatite was analyzed using scanning electron microscopy (Inspect F50, FEI, America).

### Binding potential and kinetics of PPi-Far-PMs on hydroxyapatite

Solutions of FITC-labeled PPi-Far-PMs and Far-PMs were mixed with biotechnological or commercial hydroxyapatite in round-bottom flasks (at pH 7.4). After incubation with gentle stirring for 30 min at room temperature, the mixture was filtered, washed with PBS three times, and freeze-dried. Finally, the hydroxyapatite was observed under a fluorescence microscope (FL, AMG, America).

For analysis of binding kinetics, PPi-Far-PMs or Far-PMs (1 mL) were mixed with either kind of hydroxyapatite particles (50 mg) and incubated at room temperature. At certain time points (0, 0.5, 1, 5, 30 and 720 min), hydroxyapatite was removed by centrifugation (10,000 *g,* 5 min) and the supernatant was collected. The amount of Far in the micelle supernatant after binding (W_*left*_) was analyzed by HPLC as described above. The binding rate (%) at each time point was calculated as follows: Binding rate (%) = (W_*total*_ − W_*left*_)/W_*total*_ × 100%.

### Anti-bacterial activity of Far

The ability of Far to kill *S. mutans* UA159, a proven virulent cariogenic dental pathogen, was assayed. *S. mutans* was stored at − 80 °C as stocks in brain heart infusion broth containing 25% (v/v) glycerol. These stocks were streaked onto Mitis Salivarius agar and incubated anaerobically for 48 h at 37 °C under an atmosphere of 80% N_2_, 10% H_2_ and 10% CO_2_. Then a single colony of bacteria was inoculated into brain heart infusion broth, and the culture was incubated anaerobically for 12 h at 37 °C. Bacterial growth was assayed by measurement of absorbance at 600 nm. Optical densities were converted to CFU/mL using the conversion (0.64 = 1 × 10^9^ CFU/mL [10]).

Minimum inhibitory concentration (MIC) and minimum bactericidal concentration (MBC) of Far were determined against *S. mutans* using broth microdilution [42]. The initial inoculum was 5 × 10^5^ CFU/mL, and the concentration of Far ranged from 3.5 to 448 μg/mL with twofold dilutions. Farnesol and chlorhexidine digluconate were assayed in parallel as controls. MIC was defined as the lowest concentration that showed no growth in the medium after anaerobic incubation for 48 h. Brain heart infusion broth on its own was assayed as a blank control, while the same medium supplemented with DMSO (0.076%, v/v) was assayed as a solvent control. MBC was defined as the lowest concentration that showed no surviving bacteria on the agar after incubation for 48 h at 37 °C. For MBC determination, medium in micropores without bacterial growth was picked up with a sterile inoculating loop and plated onto brain heart infusion blood agar supplemented with 5% defibrillated sheep blood. These experiments were performed three times.

### In vivo anti-caries efficacy of PPi-Far-PMs

Sprague–Dawley rats were obtained from the Laboratory Animal Center of Southwest Medical University (Luzhou China). All animal experiments were approved by the Animal Ethics Committee of this university (permit 2017060012) and carried out in accordance with the Luzhou municipal government guidelines on animal care and use.

To establish a rat model of dental caries, animals were fed a cariogenic diet with 56% sucrose (Keyes 2000; Beijing Keao Xieli Feed, Beijing China) and given 5% sucrose water to drink ad libitum [1, 43]. After weaning, 17-day-old Sprague–Dawley rats (specific pathogen-free) were fed with sodium ampicillin (0.1% in food) for 4 days to inhibit endogenous bacterial growth in the oral cavity. Any animals infected with *S. mutans* prior to inoculation were removed from the study on day 21. Then each rat was inoculated with 1 mL *S. mutans* (7 × 10^8^ CFU/mL) every day for 7 days. After being checked for infection, 29-day-old rats were randomly divided into nine groups (n = 7), and their teeth were topically treated twice daily for 5 weeks with a camel hair brush coated with the following treatments: (a) distilled water (negative control), (b) 15% ethanol (v/v, vehicle control), (c) farnesol (1.10 mg/mL), (d) farnesal (Far; 1.10 mg/mL), (e) chlorhexidine gluconate (1.10 mg/mL; positive control), (f) blank PMs, (g) Far-PMs (1.10 mg/mL Far) and (h) PPi-Far-PMs (1.10 mg/mL Far), ensuring that the drug kept working with the teeth for 1 min. All animals were weighed weekly, and physical appearance was recorded daily. At the end of the 5 weeks, saliva was collected and inoculated onto Mitis Salivarius agar with bacitracin (Sigma) to estimate the *S. mutans* population, and on brain heart infusion agar with 5% sheep blood to determine the total colony count. Finally, animals were sacrificed and the teeth were collected for further assessment.

To evaluate the anti-caries activity of PPi-Far-PMs, teeth were stained with 0.4% murexide solution, and caries were scored according to Keyes’ system [36]. A stereomicroscope (M205FA, Leica, Germany) was used to assess caries severity on the smooth-surface (Smo), proximal-surface (Pro), and sulcal-surface (Sul). Severity was graded on a four-level scale: enamel affected (E), dentin exposed (Ds), 3/4 of the dentin affected (Dm) and all dentin affected (Dx). Classifications in this study are written in the form “surface-severity”, e.g. Smo-E or Sul-Dx. The extent of caries reduction was determined using the following formula:

$${\text{Caries}}\;{\text{reduction}}\left( \% \right) = {{\left( {{\text{Keyes's}}\;{\text{score}}\;{\text{of}}\;{\text{negative}}\;{\text{control}}\;{\text{group}} - {\text{Keyes's}}\;{\text{score}}\;{\text{of}}\;{\text{test}}\;{\text{group}}} \right)} \mathord{\left/ {\vphantom {{\left( {{\text{Keyes's}}\;{\text{score}}\;{\text{of}}\;{\text{negative}}\;{\text{control}}\;{\text{group}} - {\text{Keyes's}}\;{\text{score}}\;{\text{of}}\;{\text{test}}\;{\text{group}}} \right)} {\left( {{\text{Keyes's}}\;{\text{score}}\;{\text{of}}\;{\text{negative}}\;{\text{control}}\;{\text{group}}} \right)}}} \right. \kern-\nulldelimiterspace} {\left( {{\text{Keyes's}}\;{\text{score}}\;{\text{of}}\;{\text{negative}}\;{\text{control}}\;{\text{group}}} \right)}} \times 100\%$$

Rat teeth were collected and assessed in terms of mechanical characteristics and microarchitecture. Compressive strength of teeth was measured using a universal testing machine (Meister E44, USA) at 37 ± 0.5 °C. A stress–strain curve was recorded as vertical tooth occlusal pressure was applied at 1 mm/min. After the samples were broken, the compression modulus (slope of stress–strain curve), compressive strength, compressive yield stress, and maximum stress (the highest point of stress–strain curve) were calculated base on the stress–strain curve. Microarchitecture of molars was evaluated using high-resolution micro-computed tomography (micro-CT; skyscan1172, Bruker Corporation). The following parameters were used: voltage, 80 kV; current, 80 μA; exposure time, 2.96 s; scan resolution, 14 μm/slice; and total rotation angle, 360° increasing in 0.5° increments. Bone mineral density (BMD) and bone volume per tissue volume (BV/TV) were estimated from the three-dimensional reconstructions.

### Statistical analysis

All data were expressed as mean ± standard deviation (SD) and analyzed using GraphPad Prism 6.0 (GraphPad Software, La Jolla, CA, USA). Differences between treatment groups were analyzed for significance using the Student’s *t* test or Tamhane’s T2 test. *P* < 0.05 was regarded as statistically significant.

## Supplementary information

**Additional file 1: Figure S1.**^1^H NMR and ^13^C NMR spectra of synthetic compound 1: tert-butyl 2-(2-(2-hydroxyethoxy)acetyl)hydrazine-1-carboxylate (HO-PEG-NHNH-Boc). **Figure S2.**^1^H NMR, ^13^C NMR and ^31^P NMR spectra of synthetic compound 2: sodium 2,2-dimethyl-4,7,13-trioxo-3,9,12-trioxa-5,6-diazatetradecan-14-yl hydrogen diphosphate (PPi-PEG-NHNH-Boc). **Figure S3.**^1^H NMR and ^13^C NMR spectra of synthetic compound 3: sodium 2-(2-(2-hydrazinyl-2-oxoethoxy)ethoxy)-2-oxoethyl hydrogen diphosphate (PPi-PEG-NHNH_2_). **Figure S4.**^1^H NMR and ^13^C NMR spectra of synthetic compound 4: sodium 2-oxo-2-(2-(2-oxo-2-(2-((2E,6E)-3,7,11-trimethyldodeca-2,6,10-trien-1-ylidene)hydrazinyl)ethoxy)ethoxy)ethyl hydrogen diphosphate (PPi-PEG-hyd-Far)) is available in the online version of this article.

## Data Availability

All data generated or analyzed during this study are included in this published article and its additional information files.

## Abbreviations

BHI: Brain heart infusion
BMD: Bone mineral density
Boc: t-Butyloxy carbonyl
BV/TV: Bone volume per tissue volume
CHX: Chlorhexidine digluconate
CMC: Critical Micelle Concentration
DCC: N,N′-dicyclohexylcarbodiimide
DMAP: 4-dimethylaminopyridine
Far: Farnesal
Far-PMs: Farnesal-loaded pH-sensitive polymeric micelles
Far-PMs-FITC: FITC-labeled Far-PMs
Hyd: Hydrazone bond
MBC: Minimum bactericidal concentration
MIC: Minimum inhibitory concentration
PMs: Polymeric micelles
PPi: Pyrophosphate
PPi-Far-PMs: PPi-mediated and farnesal-loaded pH-sensitive dentotropic polymeric micelles
PPi-Far-PMs-FITC: FITC-labeled PPi-Far-PMs
Pro: Proximal-surface
Pro-E: Proximal-surface with enamel affected
SEM: Scanning electron microscopy
SIS: Small intestinal submucosa
Smo: Smooth-surface
Smo-E: Smooth-surface with enamel affected
Sul: Sulcal-surface
Sul-E: Sulcal-surface with enamel affected
Sul-Dm: Sulcal-surface with 3/4 of the dentin affected
Sul-Ds: Sulcal-surface with dentin exposed
Sul-Dx: Sulcal-surface with all dentin affected
TBAP: Tris (tetra-n-butylammonium) hydrogen pyrophosphate
