# Supplementary material for: Farnesal-loaded pH-sensitive polymeric micelles provided effective prevention and treatment on dental caries
Source: J Nanobiotechnology. 2020 Jun 11;18:89. doi: 10.1186/s12951-020-00633-2 (PMC7291565; doi:10.1186/s12951-020-00633-2)
Supplement: Supplementary file 1 — Additional file 1: Figure S1.1H NMR and 13C NMR spectra of synthetic compound 1: tert-butyl 2-(2-(2-hydroxyethoxy)acetyl)hydrazine-1-carboxylate (HO-PEG-NHNH-Boc). Figure S2.1H NMR, 13C NMR and 31P NMR spectra of synthetic compound 2: sodium 2,2-dimethyl-4,7,13-trioxo-3,9,12-trioxa-5,6-diazatetradecan-14-yl hydrogen diphosphate (PPi-PEG-NHNH-Boc). Figure S3.1H NMR and 13C NMR spectra of synthetic compound 3: sodium 2-(2-(2-hydrazinyl-2-oxoethoxy)ethoxy)-2-oxoethyl hydrogen diphosphate (PPi-PEG-NHNH2). Figure S4.1H NMR and 13C NMR spectra of synthetic compound 4: sodium 2-oxo-2-(2-(2-oxo-2-(2-((2E,6E)-3,7,11-trimethyldodeca-2,6,10-trien-1-ylidene)hydrazinyl)ethoxy)ethoxy)ethyl hydrogen diphosphate (PPi-PEG-hyd-Far)) is available in the online version of this article. [file 12951_2020_633_MOESM1_ESM.docx]

# Additional Materials

## Additional table

**Table S1. Antimicrobial effect of farnesal and farnesol against *Streptococcus mutans*.**

| Compound | Minimum inhibitory concentration (μg/mL) | Minimum bactericidal concentration (μg/mL) |
| --- | --- | --- |
| Farnesal | 14 | 112 |
| Farnesol | 28 | 112 |
| Chlorhexidine Digluconate | ≤ 3.5 | 14 |

## Additional figures

### Figure S1

**Figure S1.** ^1^H NMR and ^13^C NMR spectra of synthetic compound **1**: tert-butyl 2-(2-(2-hydroxyethoxy)acetyl)hydrazine-1-carboxylate (HO-PEG-NHNH-Boc).

### Figure S2

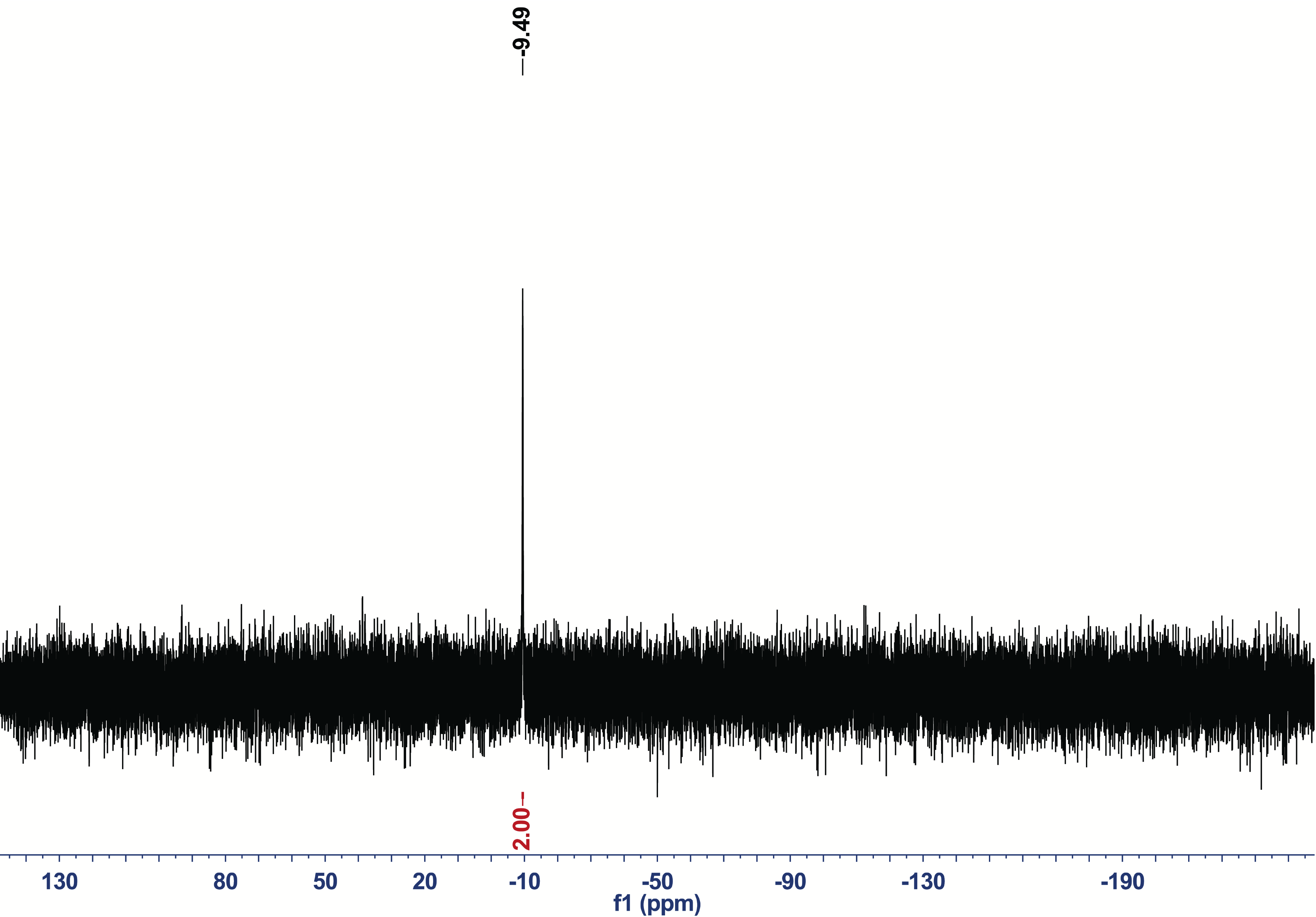


**Figure S2.** ^1^H NMR, ^13^C NMR and ^31^P NMR spectra of synthetic compound **2**: sodium 2,2-dimethyl-4,7,13-trioxo-3,9,12-trioxa-5,6-diazatetradecan-14-yl hydrogen diphosphate (PPi-PEG-NHNH-Boc).

### Figure S3

**Figure S3.** ^1^H NMR and ^13^C NMR spectra of synthetic compound **3**: sodium 2-(2-(2-hydrazinyl-2-oxoethoxy)ethoxy)-2-oxoethyl hydrogen diphosphate (PPi-PEG-NHNH_2_).

### Figure S4

**Figure S4.** ^1^H NMR and ^13^C NMR spectra of synthetic compound **4**: sodium 2-oxo-2-(2-(2-oxo-2-(2-((2E,6E)-3,7,11-trimethyldodeca-2,6,10-trien-1-ylidene)hydrazinyl)ethoxy)ethoxy)ethyl hydrogen diphosphate (PPi-PEG-hyd-Far).

### Figure S5


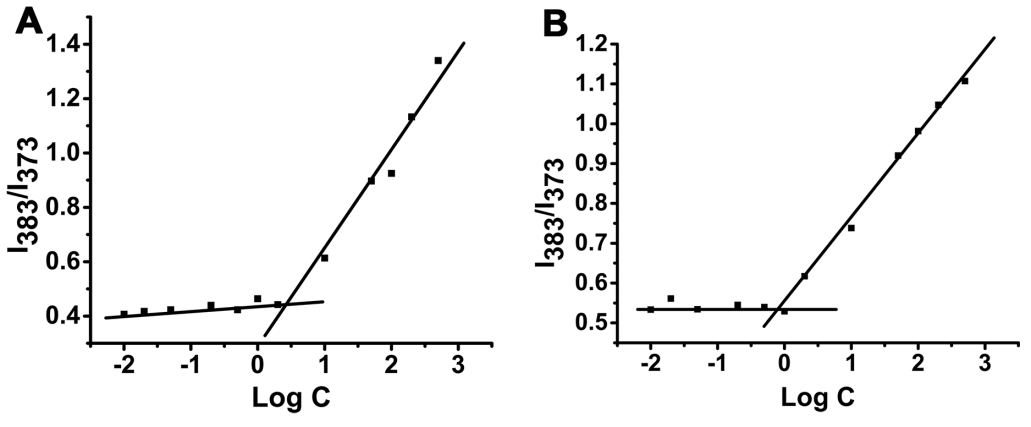


**Figure S5.** *In vitro* characterization of Blank PMs and PPi-Far-PMs. Critical micelle concentration of Blank PMs (A) and PPi-Far-PMs (B) was determined according to the concentration at the crossover point in the plots of the fluorescence intensity ratio (I_383_/I_373_) against the logarithm of micelle concentration (μg/mL).

### Figure S6


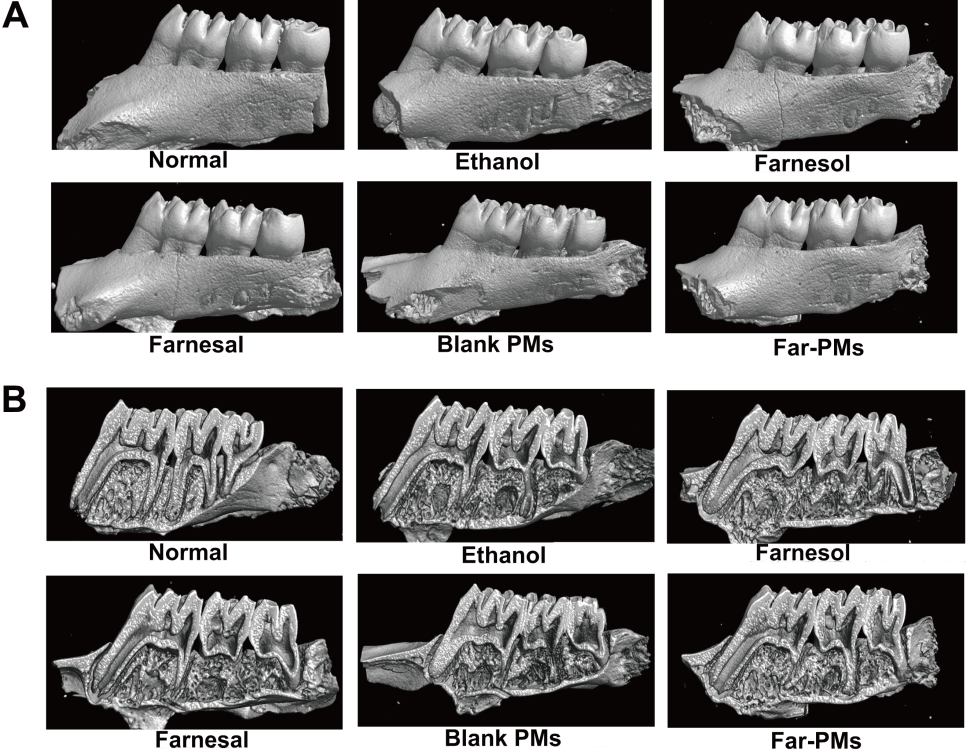


**Figure S6.** Effects of different treatments on the microarchitecture of molars in rats. Animals were treated with 15% ethanol, farnesol, farnesal, blank PMs, Far-PMs. After 5 weeks, the molars of rats were analyzed using high-resolution micro-computed tomography to obtain three-dimensional reconstruction pictures of the smooth-surface molars (A) and sulcal-surface molars (B).
